# The Effect of Pregnancy in the Hemoglobin Concentration of Pregnant Women: A Longitudinal Study

**DOI:** 10.1155/2020/2789536

**Published:** 2020-06-03

**Authors:** Berhanu Elfu Feleke, Teferi Elfu Feleke

**Affiliations:** ^1^Department of Epidemiology and Biostatistics, University of Bahir Dar, Bahir Dar, Ethiopia; ^2^Department of Pediatrics and Child Health Wolkite University, Butajira General Hospital, Ethiopia

## Abstract

**Background:**

The objective of this study was to estimate and identify the determinants of hemoglobin concentration before pregnancy, during pregnancy, and after labor and delivery.

**Methods:**

A prospective cohort study design was implemented. Data were collected from May 2015 to September 2018. A simple random sampling technique was used to select the participants. An interview technique was used to collect the data. Blood samples were collected before pregnancy, during each trimester, during labor and delivery, after third stage of labor, and at the 6-week postpartum period. Descriptive statistics were used to describe the profile of study participants. Generalized estimating equations were used to identify the determinants of hemoglobin concentration during each phase of pregnancy.

**Results:**

The mean hemoglobin concentrations of primigravida and multigravida before pregnancy were 12.41 g/dl and 10.78 g/dl, respectively. The hemoglobin concentration decreases with consecutive trimester reaching the lowest level at 42 days after delivery. The hemoglobin concentrations of pregnant women were decreased by hookworm 0.24 g/dl [95% CI:0.18-0.29], multiple pregnancy 0.16 g/dl [95% CI: 0.07-0.24], episiotomy 0.05 g/dl [95% CI: 0.01-0.09], gravidity 0.15 g/dl [95% CI: 0.09-0.21], age 0.03 g/dl [95% CI: 0.03-0.04], and gestational age 0.1 g/dl [95% CI: 0.09-0.11]. The hemoglobin concentration increased by iron supplementation 1.02 g/dl [95% CI: 0.97-1.07] and birth weight 0.14 g/dl [95% CI: 0.02-0.11].

**Conclusion:**

Pregnancy significantly decreases the hemoglobin concentration of pregnant women reaching the lowest point during labor and delivery. *Recommendation*. Regular anemia screening intervention should be implemented after delivery.

## 1. Introduction

Red blood cells are primarily formed in the bone marrow, and their production is highly affected by pregnancy. Anemia is a disease condition in which the red blood cell concentration was lower than the recommended point, a hemoglobin concentration lower than 11.6 g/dl (gram per deciliter) in the first trimester, 9.7 g/dl in the second trimester, and 9.5 g/dl in the third trimester labeled as anemia for pregnant women [1, 2].

Every day, 830 mothers die as a result of pregnancy-related complications and 98% of maternal deaths occurs in developing countries [3]. According to EDHS (Ethiopian demographic and health surveillance), the maternal mortality ratio of Ethiopia was 412 per 100,000 mothers, 45% of postpartum death occurs within the first 24 hours of delivery [4–7]. Obstetric hemorrhage is the leading cause of maternal death [8, 9]. Postpartum hemorrhage affects 14 million mothers and is responsible for the death of 125,000 mothers annually [10–13]; more than 25% of maternal death was a result of postpartum hemorrhage [14]. Postpartum hemorrhage complicates 18% of pregnancy and in resource-limited setting; the risk factors are not well identified [15].

The effects of pregnancy on the hemoglobin concentration at the different gestational age were not properly identified especially in resource-limited settings [16], so this study generates baseline information about the hemoglobin variation during each stage of pregnancy in a resource-limited setting. This study also identifies the predictors of hemoglobin concentration during each phase of pregnancy which gives important information for health professionals and decision makers.

By tackling maternal anemia with evidence-based intervention, we can prevent the occurrence of other medical complications for the mothers and newborn like congestive heart failure and renal failure [17, 18].

The objectives of this study were to estimate and identify the determinants of hemoglobin concentration before pregnancy, during pregnancy, and after labor and delivery.

## 2. Methods and Materials

A prospective cohort study design was implemented. The study was conducted in the catchment areas of Mecha Demographic Surveillance and Field Research Center, which is located in the Northwest of Ethiopia. The catchment area contains 7 rural and 3 urban kebeles with a total population of 81,000. Data were collected from May 2015 to September 2018. Initially, the survey was conducted to identify the hemoglobin concentration level of childbearing women in the district; then, update data were collected every 6 weeks. Data were collected using interview technique, reviewing the medical records, and using laboratory samples (blood and stool) collection. Blood samples were obtained from the study participants before pregnancy, during the first trimester, during the second trimester, during the third trimester, during the onset of labor, after the third stage of labor, and at the 6-week postpartum period. At each phase, 5 ml blood sample was collected from the study participant following standard operating procedures to measure the hemoglobin concentration and the red cell indices of pregnant women using a Mindray hematology analyzer. First, the blood collection procedures were clearly introduced to the study participants, a tourniquet was applied on the upper arm, and then proper antiseptic procedures were implemented; the needle was inserted on the lumen of the vein with a 15-30-degree angle with the arm surface; after taking 5 ml of blood the needle was removed and the area was pressed with a sterile gauze. A stool sample was collected from childbearing women during the baseline data collection phase. Concentration technique was used to diagnose hookworm infection. From each woman, one gram stool sample was collected in 10 ml SAF (sodium acetate-acetic acid-formalin solution). A concentration technique was used. The stool samples were well mixed and filtered using a funnel with gauze then centrifuged for one minute at 2000 RPM (revolution per minute), and the supernatant was discarded. 7 ml (milliliter) normal saline was added and mixed with a wooden stick; 3 ml ether was added and mixed well then centrifuged for 5 minutes at 2000 RPM. Finally, the supernatant was discarded, and the whole sediment was examined for parasite [19]. The source population for this study was women in the reproductive age group (15-49 years). Pregnant mothers giving birth at home were excluded. Simple random sampling technique was used to select the study participants by taking their identification number from Mecha Demographic Surveillance and Field Research Center Database.

To ensure the quality of the data, a pretest was conducted on 5% of the study participants, training was given for field workers, and the whole data collection procedures were closely supervised. Standardized operational procedures were adhered in blood and stool sample collection and analysis. The sample size was calculated using Epi-info software version 7 with the assumption of 95% CI, 85% power, 1 : 2 ratio of primigravida women to multigravida women, odds ratio of 1.5, and 10% nonresponse rate giving the final sample size of 619 primigravida women and 1321 multigravida women. The data were entered to the computer using Epi-info software and transported to SPSS software for analysis. Descriptive statistics were used to describe the profile of study participants and to estimate the mean hemoglobin concentration of women during each phase of data collection. Generalized estimating equations (GEE) were used to identify the determinants of hemoglobin concentration during each phase of pregnancy; the *B* coefficient with their 95% CI and the corresponding *P* values < 0.05 were used to declare the determinants of hemoglobin concentration.

## 3. Results

Totally, 1709 study participants were completely followed up given the response rate of 88. The mean age of the study participants was 22.58 years (SD ± 6.28 years); 79.2% of the study participants were from the rural area (Table 1).

The mean hemoglobin concentration of primigravida women before pregnancy was 12.41 g/dl, and the mean hemoglobin concentration of multigravida women before pregnancy was 10.78 g/dl. Dramatically, the hemoglobin concentration of pregnant women was declining during each trimester reaching to the lowest level during the 42 days after delivery. Iron deficiency anemia was the predominant type of anemia (Tables 2 and 3).

After adjusting for age, residence, hookworm infection, mode of delivery, multiple pregnancies, eclampsia, induction, and iron supplementation, the hemoglobin concentration was affected by hookworm, multiple pregnancy, induction, iron supplementation, episiotomy, gravidity, gestational age, birth weight, age, and preeclampsia (Table 4).

Hookworm decreases the hemoglobin concentration of pregnant women by 0.24 g/dl [95% CI: 0.18-0.29]. Multiple pregnancy decreases the hemoglobin concentration of pregnant women by 0.16 g/dl [95% CI: 0.07-0.24]. Induction increases the hemoglobin concentration of pregnant women by 0.09 g/dl [95% CI: 0.02-0.14]. Iron supplementation during pregnancy increases the hemoglobin concentration of pregnant women by 1.02 g/dl [95% CI: 0.97-1.07]. Episiotomy decreases the hemoglobin concentration of pregnant women by 0.05 g/dl [95% CI: 0.01-0.09]. The hemoglobin concentration of pregnant women decreases by 0.15 g/dl per one unit increase in gravidity [95% CI: 0.09-0.21]. Per one year increase in the age of pregnant women, the hemoglobin concentration decreases by 0.03 g/dl [95% CI: 0.03-0.04]. The hemoglobin concentration of pregnant women decreased by 0.1 g/dl per each trimester [95% CI: 0.09-0.11]. The hemoglobin concentration of pregnant women increased by 0.14 g/dl per one gram increment in the birth weight of the newborn [95% CI: 0.02-0.11] (Table 4).

## 4. Discussion

The hemoglobin concentrations of multigravida women are less than the primigravida women at the prepregnancy stage, during their pregnancy and after labor and delivery. Before pregnancy, 75% of women had a hemoglobin concentration of less than 12 g/dl; however, after the second trimester, 75% of pregnant women had a hemoglobin concentration less than 10 g/dl. Most pregnant women suffer from iron deficiency anemia (ranges from 63.9%-94.6%). This finding agrees with finding from Gondar, Ethiopia [20]. This is due to the effect of repeated exposure to pregnancy on the red blood cell formation [21]. This finding disagree with finding from China [22]; this was due to special intervention given during the pregnancy that increases the hemoglobin concentration of pregnant women in China.

Hookworm decreases the hemoglobin concentration of pregnant women by 0.24 g/dl. This finding agrees with finding from Ethiopia [23, 24]. This is due to the reason that hookworm principally ingests the red blood cells of the host [25].

Multiple pregnancies decrease the hemoglobin concentration of pregnant women by 0.16 g/dl. The mean hemoglobin concentration of singleton pregnancy is 0.16 g/dl higher than the hemoglobin concentration of multiple pregnancies. This finding agrees with finding from the United States of America [26]. Multiple pregnancies increase the volume of blood leading to hemodilution which finally decreases the hemoglobin concentration of women [26].

Induction increases the hemoglobin concentration of pregnant women by 0.09 g/dl. This finding was in line with finding from France [27]. This is due to the close supervision and additional management of women under induction that increases the hemoglobin level [28]. This finding disagrees with finding from France; this was due to the high proportion of postpartum hemorrhage in that study.

Iron supplementation during pregnancy increases the hemoglobin concentration of pregnant women by 1.02 g/dl. The mean hemoglobin concentration of women with a history of iron supplementation during pregnancy is 1.02 g/dl higher. This finding agrees with findings from India [29]. This is due to the role of iron in the red blood cell formation processes [30].

Episiotomy decreases the hemoglobin concentration of pregnant women by 0.05 g/dl. This finding was in line with a systematic review published in 2020 [31]. This is due to additional injury to the blood vessels which increases extra bleeding [32].

The hemoglobin concentration of pregnant women decreases by 0.15 g/dl per one unit increase in the gravidity; per each increment in the gravida of the women, her hemoglobin level drops by 015 g/dl. This finding agrees with finding from other parts of Ethiopia [33, 34]. This is due to the reason that the risk factors for the obstetric hemorrhage are prevalent among multigravida women [35].

Per one year increase in the age of pregnant women, the hemoglobin concentration will be dropped by 0.03 g/dl. This finding agrees with finding from the USA [36]. This is due to the fact that old age pregnancy is associated with numerous complications [37]. However, this finding disagree with South Africa research outputs [38]. This might be the higher income of women in higher age in South Africa, which means the income of women will be higher as her age increases that make them to access the different variety of foods that increases their hemoglobin concentration.

The hemoglobin concentration of pregnant women decreases by 0.1 g/dl per each trimester. This finding agrees with finding from India [39]. This is due to the reason that fetal growth and development were higher in the second and third trimesters which consumes a lot of nutrients from the mother [40].

Birth weight has a direct relationship with the hemoglobin concentration; the hemoglobin concentration of pregnant women increased by 0.14 g/dl per one gram increment in the birth weight of the newborn. This finding agrees with results from different parts of the world [41]. This is due to the good nutritional support given this group of pregnant women [42].

The main limitation of this study was a failure to detect asymptomatic cases that have an impact on the hemoglobin concentration; however, the study participants were drawn from the same community sharing more or less identical risk factors for these diseases which decreases the drawback of this study.

## 5. Conclusion

Pregnancy significantly depletes the hemoglobin concentration of the women. The lowest level of hemoglobin concentration was observed during the 6-week postpartum period. Low hemoglobin concentration is a problem for the mother even after 6 weeks of delivery.

## 6. Recommendation

Regular screening for anemia should be implemented after delivery; priority should be given for older age and women with comorbid illnesses.

## Figures and Tables

**Table 1:** Population profile of the pregnant women (*n* = 1709).

Population profile	Frequency	Percentage
Hookworm		
Infected	301	17.6
Not infected	1408	82.4
Gravidity		
Multigravida	1206	70.6
Primigravida	503	29.4
Mode of delivery		
Cesarean section	24	1.4
Instrumental delivery	111	6.5
Normal	1574	92.1
Residence		
Urban	356	20.8
Rural	1353	79.2
Multiple pregnancy		
Yes	64	3.7
No	1645	96.3
Induction		
Yes	224	13.1
No	1485	86.9
Iron supplementation		
Supplied	757	44.3
Not supplied	952	55.7
Episiotomy		
Yes	62	3.6
No	1647	96.4
Eclampsia/preeclampsia		
No	1519	88.9
Yes	190	11.1

**Table 2:** The hemoglobin concentration during the phases of pregnancy (*n* = 1709).

Visit	Mean hemoglobin concentration (g/dl)	
	Primi [95% CI]	Multi [95% CI]
Before pregnancy	12.41 [12.35-12.46]	10.78 [10.79-10.82]
During the first trimester	10.67 [10.56-10.77]	10.49 [10.46-10.52]
During the second trimester	10.11 [10.06-10.16]	10.73 [10.67-10.78]
During the third trimester	10.11 [10.06-10.16]	10.64 [10.55-10.73]
During the onset of labor	10.37 [10.26-10.19]	9.10 [9.05-9.15]
After third stage of labor	10.37 [10.26-10.19]	10.00 [9.99-10.01]
After 6 weeks	9.89 [9.77-10.01]	9.31 [9.26-9.35]

**Table 3:** The quartiles of hemoglobin concentration during each phase of pregnancy (*n* = 1709).

Visit	Mean hemoglobin concentration (g/dl)			Proportion of hypochromic, microcytic anemia
	25 percentile	50 percentile	75 percentile	
Before pregnancy	10	11	12	63.9%
During the first trimester	10	11	11	71.5%
During the second trimester	10	10	10	85.5%
During the third trimester	10	10	10	94.6%
During the onset of labor	9	10	10	91.8%
After third stage of labor	10	10	10	85.5%
After 6 weeks	9	10	10	68.2%

**Table 4:** Predictors for hemoglobin concentration (*n* = 1709).

Parameter estimates					
Variables	*B*	Standard error	95% CI (*B*)		*P* value
			Lower	Upper	
Hookworm	-0.24	0.03	-0.29	-0.18	<0.01
C/S delivery	0.02	0.07	-0.12	0.17	0.78
Instrumental delivery	-0.02	0.04	-0.09	0.06	0.69
Residence	-0.03	0.02	-0.07	0.02	0.19
Multiple pregnancy	-0.16	0.04	-0.24	-0.07	<0.01
Induction	0.09	0.03	0.02	0.14	0.01
Iron supplementation	1.02	0.03	0.97	1.07	<0.01
Episiotomy	-0.05	0.02	-0.09	-0.01	<0.01
Eclampsia	-0.09	0.04	-0.17	-0.02	0.01
Age	-0.03	<0.01	-0.04	-0.03	<0.01
Birth weight	0.14	0.02	0.11	0.17	<0.01
Gestational age	-0.1	0.01	-0.11	-0.09	<0.01
Gravidity	-0.15	0.03	-0.21	-0.09	<0.01

## Data Availability

The datasets used and/or analyzed during the current study are available from the corresponding author on reasonable request.
